# Phase I safety and pharmacokinetics of BAY 43-9006 administered for 21 days on/7 days off in patients with advanced, refractory solid tumours

**DOI:** 10.1038/sj.bjc.6602584

**Published:** 2005-05-03

**Authors:** A Awada, A Hendlisz, T Gil, S Bartholomeus, M Mano, D de Valeriola, D Strumberg, E Brendel, C G Haase, B Schwartz, M Piccart

**Affiliations:** 1 Jules Bordet Institute, Brussels, Belgium; 2 Department of Internal Medicine and Medical Oncology, West German Cancer Center, University Medical School of Essen, D-45122 Essen, Germany; 3 Bayer HealthCare, Pharma Research Center, D-42096 Wuppertal, Germany; 4 Bayer Pharmaceuticals Corporation, West Haven, CT, USA

**Keywords:** safety, pharmacokinetics, BAY 43-9006, efficacy, targeted agent, solid tumours

## Abstract

BAY 43-9006 is a novel dual-action Raf kinase and vascular endothelial growth factor receptor (VEGFR) inhibitor that targets tumour cell proliferation and tumour angiogenesis. This Phase I study was undertaken to determine the safety profile, maximum tolerated dose (MTD), dose-limiting toxicities (DLTs), pharmacokinetics, and tumour response profile of oral BAY 43-9006 in patients with advanced, refractory solid tumours. BAY 43-9006 was administered daily for repeated cycles of 21 days on/7 days off. A total of 44 patients were enrolled at doses from 50 to 800 mg b.i.d. Pharmacokinetic profiles of BAY 43-9006 in plasma were determined during the first treatment cycle. The most frequently reported adverse events over multiple cycles were gastrointestinal (75%), dermatologic (71%), constitutional (68%), pain (64%), or hepatic (61%) related. A MTD of 400 mg b.i.d. BAY 43-9006 was defined. BAY 43-9006 was absorbed rapidly; steady-state conditions were reached within 7 days. BAY 43-9006 exposure increased nonproportionally with increasing dose. In all, 32 patients were evaluated for tumour response: 15 patients showed tumour progression, 16 patients experienced stable disease (>6 months in eight patients), and one patient with renal cell carcinoma achieved a partial response. BAY 43-9006 given for 21 days with 7 days off treatment was safe, well tolerated, and showed antitumour activity.

Activation of receptor tyrosine kinases (RTKs) by cytokines or growth factors initiates a protein-kinase-mediated cascade of molecular events that ultimately leads to control of cell growth and survival. The growth factor receptors activate the cellular Ras proteins, which interact with and activate the Raf protein kinase. The Raf protein kinase activates the Raf/MEK/ERK pathway that initiates cell proliferation. Overactivation of this pathway has been observed in many human malignancies and, therefore, components of this cascade are attractive targets for anticancer therapies (Hoshino *et al*, 1999; Avruch *et al*, 2001).

Raf proteins are not solely key downstream effector molecules of Ras; recent work suggests a direct role for Raf in the development and maintenance of cancer. Overexpression of constitutively active c-Raf is sufficient for NIH 3T3 cellular transformation (Kerkhoff and Rapp, 1997). Mutations in the *BRAF* gene derived from human cancer cell lines and human primary cancers can affect cellular transformation of NIH 3T3 cells, and Raf can exert tumorigenic effects independent of Ras (Davies *et al*, 2002; Rajagopalan *et al*, 2002). Furthermore, it has been suggested that Raf signalling is necessary for tumour neovascularisation, which is essential for tumour survival, growth, and metastasis (Bergers *et al*, 2003).

BAY 43-9006 is a potent, small-molecule inhibitor of c-Raf and wild-type and mutant V599E B-Raf (Wilhelm *et al*, 2004). BAY 43-9006 inhibits c-Raf-mediated ERK phosphorylation *in vitro* and decreases endogenous phosphorylated ERK in tumour cell lines containing either mutant Ras or mutant B-Raf. BAY 43-9006 also targets the vascular endothelial growth factor (VEGF) receptors, VEGFR-2 and VEGFR-3, and platelet-derived growth factor receptor-*β* (PDGFR-*β*) – key RTKs involved in tumour progression and angiogenesis (Wilhelm *et al*, 2004).

In human tumour xenograft models, BAY 43-9006 significantly delayed tumour growth of colon, pancreatic, and non-small-cell lung cancer (NSCLC) models containing either B-Raf or K-Ras mutations (Wilhelm *et al*, 2004). BAY 43-9006 also slowed tumour growth of ovarian cancer derived from the SKOV-3 cell line, which contains wild-type K-Ras but overexpresses epidermal growth factor and HER-2 receptors (Gianpaolo-Ostravage *et al*, 2001; Lyons *et al*, 2001). Administration of BAY 43-9006 to mice with human colon tumours (HT-29 or Colo-205) or MBA-MD-231 human breast tumour xenografts significantly reduced tumour neovascularisation, as measured by decreased CD31-positive microvessel density (Wilhelm *et al*, 2004).

The anticancer activity of BAY 43-9006 appears to be cytostatic, requiring continued drug exposure for tumor growth inhibition. In preclinical studies, tumor growth was inhibited in the presence of the drug, but returned to baseline growth rates upon treatment cessation. In order to achieve a balance between minimising time off BAY 43-9006 and avoiding potential toxicity associated with extended exposure, four Phase I studies were initiated to determine the most favorable and effective dosing regime (Strumberg *et al*, 2003).

This Phase I trial was conducted to determine the dose-limiting toxicity (DLT), maximum tolerated dose (MTD), and the safety and pharmacokinetics of BAY 43-9006 administered for 21 consecutive days, followed by 7 days off treatment, in patients with refractory solid tumours.

## PATIENTS AND METHODS

### Patient selection

A total of 44 patients with advanced, refractory solid tumours were enrolled between December 2000 and December 2002. Eligible patients were at least 18 years of age and had failed curative measures, an Eastern Cooperative Oncology Group (ECOG) performance status of ⩽2 and a life expectancy of ⩾12 weeks. These patients also had adequate bone marrow (haemoglobin ⩾10 mg dl^−1^, platelets >100 000 mm^−3^, neutrophils ⩾2000 mm^−3^), hepatic (bilirubin ⩽1.5 × upper limit of normal (ULN), alanine aminotransferase (ALT), and aspartate aminotransferase (AST) ⩽5 × ULN), and renal (serum creatinine ⩽1.5 × ULN or calculated creatinine clearance >60 ml min^−1^ function.

Patients with primary or metastatic brain or meningeal tumours were excluded from the study, as were patients with clinically evident congestive heart failure, serious cardiac arrhythmias, or symptoms of coronary heart disease or ischaemia. Patients with serious active infections, and pregnant or lactating women were also excluded from the study. Enrolled patients had not received immunotherapy or chemotherapy within 4 weeks of study entry, or radiotherapy within 3 weeks of study entry.

All patients received information on the purpose and conduct of this study, and provided written, informed consent in accordance with federal and institutional guidelines. This study received institutional ethical approval and was conducted in accordance with the Declaration of Helsinki and good clinical practice.

### Treatment plan

This was an open-label, noncontrolled, single-centre (Brussels, Belgium) dose-escalation study in which groups of three to six patients received oral BAY 43-9006 administered in repeated cycles of 21 days on drug followed by a 7-day rest period. The first two groups received 50 mg BAY 43-9006 once daily – either 50 mg every 5 days, or 50 mg every other day (henceforth referred to as 50 mg combination (COMB)). The remaining groups received BAY 43-9006 twice daily (b.i.d.) at a dose of 100, 200, 300, 400, 600, or 800 mg for 21 consecutive days.

**Figure 1:**
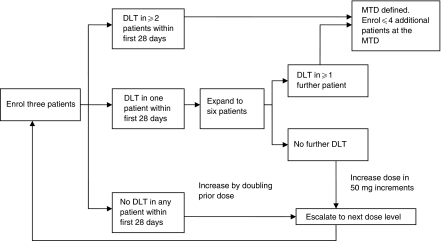
Dose-escalation schema.

Three patients were initially enrolled in each group (Figure 1). Groups were treated according to a standardised dose-escalation scheme, depending on the worst toxicity experienced by patients enrolled at the prior dose level, and the clinical significance of that toxicity. In the absence of DLT within the first 28 days, the next group of three patients was enrolled at the next dose level. If DLT was observed in at least two patients enrolled in a given group within the first 28 days, the MTD was defined as the previous dose level. If only one patient experienced DLT, a further three patients were enrolled in that group. If DLT was observed in at least one more of these six patients, the MTD was defined as the previous dose level. If none of the additional patients in this expanded cohort experienced a DLT, dose escalation continued. Once the MTD was established, up to four additional patients could be enrolled at the MTD level (up to 10 patients in total) to obtain additional safety data at the recommended dose.

Adverse events (AEs) possibly or probably related to the study drug were considered DLTs if they met any of the following criteria: nonhaematological toxicity (excluding alopecia) of at least grade 3 severity according to version 2.0 of the National Cancer Institute Common Toxicity Criteria (NCI-CTC); neutropenia of NCI-CTC grade 4 lasting for ⩾4 days; thrombocytopenia of at least NCI-CTC grade 3. Patients in any cohort who experienced a DLT before the end of a treatment cycle had the number of DLT-free days recorded. Following the appearance of a DLT, study medication was withheld until toxicity resolved or returned to a severity ⩽grade 2, or until the principle investigator considered it appropriate to resume treatment. The recovery period was also noted for each patient. Patients resumed BAY 43-9006 at the initial dose, but an intermittent dosing schedule was utilised, depending on the duration of DLT-free days and the DLT recovery time, based on an algorithm. If patients experienced a DLT on this intermittent schedule, BAY 43-9006 was withheld until recovery, after which time the patient received BAY 43-9006 at the next lower dose level.

### Patient evaluation

Pretreatment evaluations took place 7 days before enrolment, and included physical examinations (weight, height, vital signs), ECOG performance status, documentation of all measurable or evaluable disease, recording of concurrent medications, routine laboratory evaluations (complete blood counts, platelets, differential counts, biochemistry, and clotting times), and pregnancy tests for women. Pretreatment studies also included an electrocardiogram (ECG) within the 28 days before enrolment.

Physical examinations were performed on day 1 of each treatment cycle, haematological laboratory evaluations twice weekly during the treatment period, and biochemical evaluations twice weekly during the first cycle, and twice per cycle thereafter. Clinical tumour measurements were carried out as part of the physical examination. Scans and X-rays to document the disease were obtained every 8 weeks, and were compared with those obtained prior to treatment. Tumour response was evaluated according to the Response Evaluation Criteria in Solid Tumours (RECIST) system (Therasse *et al*, 2000). Complete or partial responses (CR or PR) required a confirmatory scan at least 4 weeks later. For an assessment of stable disease (SD), documentation of SD was required at least once ⩾4 weeks from baseline.

### Safety

All patients who received at least one dose of BAY 43-9006 were included in the safety analysis. Adverse events were assessed at the end of each cycle and up to 30 days after the last dose of study medication, and were graded according to the NCI-CTC version 2 (Trotti *et al*, 2000). Patients with grade 3/4 treatment-related toxicities were required to improve to ⩽grade 2 before resuming treatment. If tumour progression occurred, treatment with the study medication was discontinued, and a final visit took place within 2 weeks.

### Pharmacokinetics

Patients who completed at least one cycle of BAY 43-9006 and had no missing pharmacokinetic measurements were valid for pharmacokinetic analysis. Blood samples from patients in the 100 mg b.i.d. group or higher were collected on days 1, 7, and 21 of the first cycle. Blood samples (2 ml aliquots) were collected at 0 (predose), 2, 3, 4, 6, 12, and 24 h after study drug administration. When BAY 43-9006 was given b.i.d., blood was collected up to 12 h after administration. After the last dosage on day 21, two additional samples were collected 48 and 96 h later.

Plasma was removed from the blood samples following centrifugation, and stored at −70°C. A validated LC-MS/MS method with multiple reaction monitoring was used for determination of BAY 43-9006 plasma concentration with lower limits of quantification (LOQ) at 1.0, 2.4, or 100 *μ*g l^−1^ depending on the method used. Method validation was performed in accordance with internationally accepted requirements. Mean accuracy for quality control samples ranged from 91.7 to 104.9%, and mean precision from 0.7 to 13.5% for all methods used.

Plasma pharmacokinetic parameters – area under the curve (AUC), maximum concentration (*C*_max_), time to maximum concentration (*t*_max_), and apparent terminal half-life (*t*_1/2_) – for BAY 43-9006 were calculated by noncompartmental methods using KINCALC (a program developed by Bayer HealthCare). This program uses a log-linear trapezoidal method for determining the AUC values. Pharmacokinetic parameters were analysed with descriptive statistics. Plasma concentration–time courses of BAY 43-9006 (calculated if two-thirds or more of the individual values were above the LOQ) are presented as geometric means.

## RESULTS

### Patients’ characteristics

Patients received BAY 43-9006 50 mg once every 5 days or every other day (seven patients), or 100 mg b.i.d. (four patients), 200 mg b.i.d. (three patients), 300 mg b.i.d. (five patients), 400 mg b.i.d. (10 patients), 600 mg b.i.d. (12 patients), and 800 mg b.i.d. (three patients) for 21 consecutive days followed by 7 days off treatment. Patients’ baseline characteristics are shown in Table 1. All patients on study were Caucasian. The most common forms of cancer were colon carcinoma (34%), breast cancer (16%), and kidney carcinoma (16%); all patients presented with metastatic cancer. Before study entry, all patients had been treated with systemic therapy, 41 (93%) with surgery and 22 (50%) with radiotherapy (Table 1). Grade 3 hepatic abnormalities were recorded at baseline in one patient each from the 100 mg b.i.d., 200 mg b.i.d., 300 mg b.i.d., and 600 mg b.i.d. groups, and in three patients in the 400 mg b.i.d. group. Five of these patients had liver metastases at baseline.

### Patient outcomes

A total of 42 (95%) patients discontinued the study, most commonly because of disease progression (68%) or AEs (18%). All nine patients who discontinued because of AEs were from the ⩾300 mg b.i.d. groups. Of these nine patients, only one discontinued as a result of a serious event (oedema of the uvula) related to study medication (600 mg b.i.d. dose group). Six (14%) patients died while on study medication due to tumour progression; none of the events resulting in death was considered treatment related.

### Dose escalation and modification

No nonhaematological DLT occurred at the 50 or 100 mg b.i.d. dose levels. One of three patients (33%) developed DLT at the 800 mg b.i.d. dose level, and seven of 12 patients (58%) enrolled at 600 mg developed DLT. An additional group treated with BAY 43-9006 400 mg b.i.d. was examined because of the high proportion of serious drug-related AEs in the 600 mg b.i.d. group, and the lack of ⩾grade 3 toxicities in the 300 mg b.i.d. group (except one patient with fatigue). Of the first three patients treated at 400 mg b.i.d., one had drug-related toxicity ⩾grade 3. Three additional patients were enrolled in this group, and no DLTs were observed, so the group was increased to 10 patients.

### Treatment and dose exposure

The overall median duration of treatment with BAY 43-9006 was 42 days (Table 2). The duration of treatment varied widely, ranging from 1 to 261 days of treatment. The greatest time on study drug was in the 600 mg b.i.d. (starting dose) group: >50% of patients received more than three cycles, for a median treatment duration of 95 days (Table 2). Of note, the dose for nine of 12 (75%) of these patients treated at the starting dose of 600 mg b.i.d. was reduced to 400 mg b.i.d. until the end of therapy. Of these nine patients, six (67%) experienced DLTs before their dose was reduced to 400 mg b.i.d. BAY 43-9006. Two of the three patients assigned to 800 mg b.i.d. received a dose reduction: one patient to 600 mg b.i.d. 5 days after treatment initiation and prior to the patient being discontinued due to no clinical benefit (total one cycle); one patient to 600 mg b.i.d. (four cycles) and then to 400 mg b.i.d. (five cycles) due to toxicity (total of 10 cycles received).

### Safety

All 44 patients were evaluated for safety and AEs were reported for all patients. The most frequently reported treatment-emergent toxicities over multiple cycles were gastrointestinal (75% of patients), dermatologic/skin disorders (71%), constitutional symptoms (68%), pain (64%), and hepatic disorders (61%). Individual AEs occurring in ⩾20% of patients were anorexia, nausea, diarrhoea, and stomatitis/pharyngitis for the gastrointestinal toxicities; hand–foot skin reaction (HFS), rash/desquamation, pruritus, and alopecia for dermatological toxicities; and fatigue and fever for constitutional symptoms. Other pain (excluding abdominal, bone or tumour pain, headache, arthralgia, or myalgia) occurred in 30% of patients, and abnormal AST, ALT, alkaline phosphatase (ALP), and bilirubin levels were the most frequent hepatic-related events (occurring in ⩾20% of patients).

A total of 34 patients experienced drug-related AEs of any grade; the most common AEs were fatigue (52% of patients), HFS (43%), anorexia (39%), and rash/desquamation (37%). Of these drug-related AEs, 13 (30%) were ⩾grade 3. At the 400 mg b.i.d. dose level, the most common drug-related AEs were skin-related and hepatic disorders – HFS (50%), rash/desquamation (60%), pruritus (30%), alopecia (30%), abnormal AST (50%), ALT (50%), ALP (30%), and bilirubin (40%). In general, drug-related events in this group did not exceed grade 2 toxicity (Table 3). One patient in this group experienced grade 3 fatigue, nausea, anorexia, vomiting, and pain. All patients in the 600 mg b.i.d. dose group experienced drug-related AEs, of which seven (58%) were ⩾grade 3.

Haematological toxicities (abnormal haemoglobin, total white blood count, and platelets) were mild to moderate in severity, and were not dose limiting. Negligible effects on granulocytes were observed. No patient showed any change > grade 2 in these parameters from baseline, except one patient in the 50 mg COMB group, whose haemoglobin changed several times from grade 1 at baseline to grade 3 during cycles 3–7.

In the higher dosage groups, more AEs were drug related and led to permanent discontinuation of treatment. The incidences of fatigue, fever without neutropenia, HFS, rash/desquamation, pruritus, diarrhoea, stomatitis/pharyngitis, sensory neuropathy, and elevated AST, ALT, and ALP were increased in the groups receiving >300 mg b.i.d. BAY 43-9006.

One of the most frequently observed AEs was HFS, which was reported in 19 (43%) patients. Grade 3 HFS occurred in five (42%) patients in the 600 mg b.i.d. group. A review of patients’ medical histories indicated that four patients had received prior anticancer therapy with agents known to frequently cause HFS (liposomal doxorubicin and/or capecitabine).

### Maximum tolerated dose and DLT

A total of 11 patients experienced DLT: one patient each from the 50 mg COMB, 300, 400, and 800 mg b.i.d. groups, and seven patients from the 600 mg b.i.d. group. Dose limiting toxicities of grade 3 dyspnoea and fatigue were reported for one of three patients receiving 800 mg b.i.d. BAY 43-9006. Seven (58%) patients receiving 600 mg b.i.d. experienced DLTs, including HFS (three patients, two of whom also had generalised rash), and abdominal cramping, diarrhoea, retrosternal pain, oedema of uvula, anorexia. At 400 mg b.i.d., only one out of 10 patients developed DLT (grade 3 fatigue, anorexia, vomiting, nausea, pain). Owing to the unacceptable incidence rate of DLTs at the 600 mg b.i.d. dose, 400 mg b.i.d. BAY 43-9006 was determined to be the MTD, and is the recommended dose for Phase II studies.

### Pharmacokinetics

**Figure 2:**
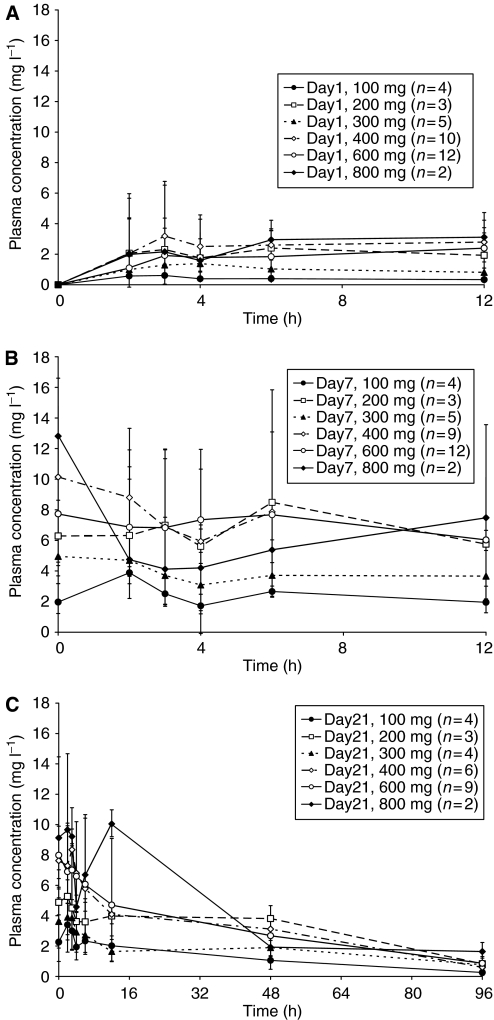
Mean plasma concentrations of BAY 43-9006 on days 1 (**A**), 7 (**B**), and 21 (**C**).

Plasma concentration–time profiles of patients in the 100 mg b.i.d. group and higher are shown in Figure 2. BAY 43-9006 is absorbed rapidly after the first dose on day 1, and peak concentrations in plasma occurred within 2–7 h and were maintained at 12 h postdose (Figure 2A). After multiple doses of BAY 43-9006, mean plasma concentrations were highly variable and showed no clear dose dependency. Times of peak concentrations ranged from 0 h (before dosing) to 12 h (Figure 2B and C).

Mean AUC_0–12_ and *C*_max_ for BAY 43-9006 following doses of 200, 400, and 600 mg b.i.d. on days 1 (first dose), 7, and 21 are listed in Table 4. For the MTD of 400 mg b.i.d., mean AUC_0–12_ was 24.0 mg h l^−1^ on day 1, increased to 82.7 mg h L^−1^ by day 7 and declined slightly to 76.5 mg h l^−1^ by day 21. Similarly, mean *C*_max_ for the 400 mg b.i.d. dose increased substantially between days 1 and 7 (from 3.04 to 9.90 mg l^−1^), with little change between days 7 and 21 (Table 4). Mean AUC_0–12_ and *C*_max_ values for the other dose levels also did not appear to increase further after day 7 (Table 4). The terminal half-life (*t*_1/2_) could be determined following the last dose on day 21. Mean t_1/2_ ranged from 24 to 39 h (Table 4). Comparison of the accumulation ratios for *C*_max_ and AUC_0–12_ (*R*_A1_ and *R*_A3_, respectively) indicates that steady-state conditions were reached after 7 days of dosing. Mean *R*_A1_ and *R*_A3_ values after continuous dosing with ⩾100 mg b.i.d. BAY 43-9006 ranged from 2.6 to 6.2, and from 2.5 to 6.4, respectively, on Day 7, and ranged from 2.0 to 6.3, and from 1.7 to 6.7, respectively, on day 21.

### Response to therapy

Tumour response was evaluated in 32 (73%) patients; tumour response evaluations were either not done or missing in six patients, and an additional six patients were not evaluable. Of the patients evaluable for efficacy, one patient experienced a PR, and 16 patients (50%) experienced SD as best response (Table 5). Of the patients with SD, two had SD >12 months (600 mg b.i.d. starting dose group) and six had SD of 6–12 months (300 mg b.i.d. (*n*=1); 400 mg b.i.d. (*n*=1); 600 mg b.i.d. (*n*=3); 800 mg b.i.d. starting dose group (*n*=1)). In all cases, patients with SD of long duration were switched to 400 mg b.i.d. during the period of SD, which was established as the MTD. A total of 26 (59%) patients showed tumour progression at some time during the study, and for 15 of these patients, the best response recorded was progressive disease (Table 5). In total, 19 (43%) patients survived at least 6 months following the first dose of BAY 43-9006, and three (7%) patients were alive at least 12 months after the first dose was administered.

Of the three patients assigned to BAY 43-9006 800 mg b.i.d., two patients withdrew from the study as a result of insufficient therapeutic effect and toxicities. The third patient had a dose reduction following the development of AEs and experienced SD as a best response that lasted for a median of 276 days.

Although patients who began treatment in the 600 mg b.i.d. group tended to have a longer median time to progression, they were more likely to be treated for more than three cycles, and a higher proportion of PR/SD best response assessments compared with the other groups, six of the eight PR/SD patients required a dose reduction to 400 mg b.i.d. during the treatment period. One patient with renal cell carcinoma (RCC) who received the starting dose of 600 mg b.i.d. achieved a PR lasting a total of 104 days, during which the patient received 400 mg b.i.d. for 99 days.

## DISCUSSION

The results of this Phase I trial show that oral BAY 43-9006 is safe and well tolerated when administered for 21 consecutive days followed by a 7-day rest period. Adverse events were generally mild to moderate and manageable with supportive medications. The most common drug-related toxicities were fatigue, HFS, and rash. Significant neutropenia, thrombocytopenia, and anaemia were not observed during treatment with BAY 43-9006, suggesting that BAY 43-9006 can be combined with other anticancer agents. In addition, life-threatening CNS, cardiac, hepatic, and renal toxicities were not observed. A dose-dependent relationship was observed for certain AEs, with a higher incidence of fatigue, HFS, rash/desquamation, pruritus, diarrhoea, stomatitis/pharyngitis, and sensory neuropathy in the higher dose groups (>300 mg b.i.d.). The 400 mg b.i.d. dose was established as the MTD after obtaining an unacceptable incidence rate of DLT at 600 mg b.i.d.

Interestingly, the AE findings in this study are similar to those of three other parallel Phase I trials with BAY 43-9006, which examined various dosing schedules (continuous b.i.d. dosing, 28 days on drug/7 days off, and 7 days on drug/7 days off) treating a wide range of tumour types (Strumberg *et al*, 2003). Similar to the results presented, the 400 mg b.i.d. dose had the most favourable toxicity profile of the higher dose levels evaluated, and data from the entire Phase I programme identify 400 mg b.i.d. BAY 43-9006 continuously as the recommended dose for Phase II studies (Strumberg *et al*, 2003).

The pharmacokinetic profile in this study showed that BAY 43-9006 b.i.d. is orally bioavailable in cancer patients with solid tumours refractory to standard care. Pharmacokinetic findings for the BAY 43-9006 dosing schedule reported here are consistent with those obtained in the three other Phase I studies (Strumberg *et al*, 2003). BAY 43-9006 is rapidly absorbed after the first dose, and the t_1/2_ after multiple dosing of 200–600 mg b.i.d. ranged from 24 to 39 h. Steady-state conditions were achieved after 7 days of dosing, with no further accumulation after an additional 14 days of b.i.d. dosing. These pharmacokinetic findings support 400 mg b.i.d. as the recommended Phase II dose.

Following treatment with BAY 43-9006, 50% of patients experienced SD. Treatment with a starting dose of 600 mg b.i.d., with a dose reduction to 400 mg b.i.d., due to toxicities at 600 mg b.i.d. dose, was associated with the most favourable response profile. Of the 16 patients with SD, duration of SD was 6–12 months in six patients, and >12 months in two patients (one with colorectal cancer and the other patient with head and neck cancer). Another patient with RCC experienced a PR lasting 104 days. All five patients with prolonged SD began dosing at 600 or 800 mg b.i.d. and rapidly underwent dose reduction to 400 mg b.i.d. during the period of SD.

Renal cell carcinoma tumours are well vascularised, and recent work links this type of cancer with mutations in the von Hippel–Lindau tumour suppressor gene, which ultimately leads to the overproduction of VEGF and a more aggressive tumour phenotype (Grugel *et al*, 1995). BAY 43-9006 directly inhibits two VEGF receptors, VEGFR-2 and VEGFR-3. BAY 43-9006 also targets both Raf-1 and B-Raf kinase proteins, which promote proliferation through the Ras mitogen pathway, and are also implicated in suppressing apoptosis and supporting angiogenesis (Wang *et al*, 1996; Erhardt *et al*, 1999; Schulze *et al*, 2001; Zhong *et al*, 2001). Mice lacking these genes exhibit increased apoptosis and significant vascular defects (Wojnowski *et al*, 1997; Huser *et al*, 2001).

BAY 43-9006 is a novel Raf kinase and VEGFR inhibitor that targets two mechanisms of tumour development – tumour cell proliferation and tumour angiogenesis. In this Phase I trial, BAY 43-9006 was well tolerated, with a manageable safety profile. The MTD was determined as 400 mg b.i.d., which is being used in Phase II studies to further assess safety and efficacy of BAY 43-9006 in a range of tumour types. A Phase III study is under way in patients with advanced RCC.

## Figures and Tables

**Table 1:** Demographic and clinical characteristics of patients treated on study

**Characteristic**	**Number of patients**
No. of patients (evaluable)	44
Median age (range), years	58 (42–79)
Gender (male : female)	25 : 19

*ECOG performance status (n* (%))	
0	12 (27)
1	30 (68)
2	2 (5)

*Previous therapy (n* (%))	
Surgery	41 (93)
Systemic therapy	44 (100)
Radiotherapy	22 (50)

*Tumour types*	
Colon	15 (34)
Breast	7 (16)
Kidney	7 (16)
Ovary	1 (2)
Liver	1 (2)
Gastrointestinal (other)	2 (5)
Head and Neck	1 (2)
Lung	1 (2)
Melanoma	2 (5)
Unknown	5 (11)
Other	2 (5)

**Table 2:** Exposure to BAY 43-9006 in patients with advanced, refractory solid tumours

	**Starting dose of BAY 43-9006**							
	**50 mg COMB^a^ (*n*=7)**	**100 mg b.i.d. (*n*=4)**	**200 mg b.i.d. (*n*=3)**	**300 mg b.i.d. (*n*=5)**	**400 mg b.i.d. (*n*=10)**	**600 mg b.i.d.^b^ (*n*=12)**	**800 mg b.i.d. (*n*=3)**	**Total (*n*=44)**
Median number of 28-day treatment cycles administered (range)	2 (1–12)	2.5 (2–4)	2 (1–2)	2 (1–6)	3 (1–9)	5 (2–14)^b^	1 (1–10)	
No. of patients receiving more than three cycles (*n* (%))	1 (14)	1 (25)	0 (0)	1 (20)	3 (30)	7 (58)^b^	1 (33)	
Median duration of treatment, days (range)^c^	42 (11–252)	50 (42–84)	42 (21–48)	42 (21–104)	48 (1–157)	95 (11–261)^b^	21 (13–175)	42 (1–261)

a Combination of two different 50 mg presentations of study drug.

b Nine of these patients were reduced to 400 mg b.i.d. due to toxicity.

c Calculated by adding 21 days of study drug treatment for each completed 28-day cycle.

**Table 3:** Incidence of drug-related adverse events occurring in >10% of patients in patients with advanced, refractory solid tumours

	**50 mg COMB (*n*=7)**		**100 mg b.i.d. (*n*=4)**		**200 mg b.i.d. (*n*=3)**		**300 mg b.i.d. (*n*=5)**		**400 mg b.i.d. (*n*=10)**		**600 mg b.i.d. (*n*=12)**		**800 mg b.i.d. (*n*=3)**		**Total (*n*=44)**	
	**Grade (%)**		**Grade (%)**		**Grade (%)**		**Grade (%)**		**Grade (%)**		**Grade (%)**		**Grade (%)**		**Grade (%)**	
	**1/2**	**3/4**	**1/2**	**3/4**	**1/2**	**3/4**	**1/2**	**3/4**	**1/2**	**3/4**	**1/2**	**3/4**	**1/2**	**3/4**	**1/2**	**3/4**
Blood/bone marrow		14	25								33				14	2
Fatigue	14		25				40	20	40	10	75	8	67	33	43	9
Hand–foot skin reaction	14						40		50		41	42	33		32	11
Rash/desquamation	14								60		50	17	33		32	5
Pruritus	14						40		30		50	8	67		32	2
Alopecia	14		25		33		20		30		50				30	
Dry skin					33						33				11	
Anorexia	14		25		67		60		20	10	34	8	67		34	5
Diarrhoea							20		10		42			33	16	2
Stomatitis/pharyngitis							20		20		33			33	16	2
Nausea					33					10	17		67		11	2
AST							40		50		50		33		32	
ALT									50		25		33		21	
ALP									30		42				18	
Bilirubin									40				33		11	
Sensory neuropathy							20		20		17		33		14	
Other pain^a^					33				20	10	17		33		14	2

Adverse events were rated according to National Cancer Institute Common Toxicity Criteria (NCI CTC) Version 2.0.

ALP=alkaline phosphatase; ALT=alanine aminotransferase; AST=aspartate aminotransferase.

a Other pain excluded arthralgia, myalgia, abdominal pain and cramping, bone pain, tumour pain, and headache.

**Table 4:** Mean pharmacokinetic parameters for BAY 43-9006 following the first dose (day 1) and multiple (days 7 and 21) b.i.d. oral dosing (geometric means, geometric standard deviations)

**Pharmacokinetic parameter**	**BAY 43-9006 dose b.i.d. (mg)**	***n***	**Mean (standard deviation)**	**Range**
*Day 1*				
AUC_0–12_ (mg h l^−1^)	200	3	24.9 (1.39)	17.3–32.6
	400	9	24.0 (1.51)	13.9–46.4
	600	12	30.4 (1.68)	9.02–65.0
*C*_max_ (mg l^−1^)	200	3	3.63 (1.26)	3.00–4.70
	400	10	3.04 (1.89)	0.92–7.55
	600	12	4.56 (1.69)	1.58–12.1

*Day 7*				
AUC_0–12_ (mg h l^−1^)	200	3	83.4 (1.44)	64.2–126
	400	9	82.7 (1.62)	39.6–201
	600	12	94.8 (1.62)	45.1–199
*C*_max_ (mg l^−1^)	200	3	9.01 (1.80)	5.58–17.4
	400	9	9.90 (1.52)	5.63–23.0
	600	12	11.5 (1.54)	5.96–21.8

*Day 21*				
AUC_0–12_ (mg h l^−1^)	200	3	50.5 (1.14)	45.2–58.4
	400	5	76.5 (1.28)	60.1–112
	600	6	77.0 (1.55)	46.8–160
*C*_max_ (mg l^−1^)	200	3	6.33 (1.61)	4.79–11.0
	400	6	10.0 (1.26)	7.46–15.1
	600	8	9.24 (1.53)	4.72–16.7
*t*_1/2_ (h)	200	2	29.8 (1.14)	27.2–32.8
	400	3	23.8 (1.45)	16.7–35.2
	600	6	38.6 (1.45)	24.1–72.3

**Table 5:** RECIST (Response Evaluation Criteria in Solid Tumours)-defined best response in patients with advanced, refractory solid tumours receiving BAY 43-9006

	**Starting dose of BAY 43-9006**							
	**50 mg COMB (n=7)**	**100 mg b.i.d. (*n*=4)**	**200 mg b.i.d. (*n*=3)**	**300 mg b.i.d. (*n*=5)**	**400 mg b.i.d. (*n*=10)**	**600 mg b.i.d. (*n*=12)**	**800 mg b.i.d. (*n*=3)**	**Total (*n*=44)^a^**
Best response assessed	5	4	2	4	7	9	1	32/44 (73%)
*Best response*								
Partial response						1		1/32 (3%)
Stable disease (all)	2	2		1	3	7	1	16/32 (50%)
6–12 months				1	1	3	1	6/32 (19%)
>12 months						2		2/32 (6%)
Progressive disease	3	2	2	3	4	1	0	15/44 (59%)
Time to progression								
Median, days (range)	70 (50–86)	65 (55–107)	52 (51–52)	44 (22–106)	63 (24–123)	155 (56–280)	276	69 (22–280)

a Tumour response was evaluable in 32 patients.
